# Health seeking behaviours, dengue prevention behaviours and community capacity for sustainable dengue prevention in a highly dengue endemic area, Sri Lanka

**DOI:** 10.1186/s12889-023-15404-5

**Published:** 2023-03-16

**Authors:** R. M. Nayani Umesha Rajapaksha, Chrishantha Abeysena, Aindralal Balasuriya

**Affiliations:** 1 University of Colombo, Colombo, Sri Lanka; 2 University of Kelaniya, Ragama, Sri Lanka; 3 General Sir John Kotelawala Defence University, Ratmalana, Sri Lanka

**Keywords:** Dengue, Vector-born disease, Endemic, Behaviours, Community capacity, Prevention

## Abstract

**Introduction:**

Dengue has become a major health problem in globally as well as locally. The delay in health-seeking is significantly associated with complications leading to severe dengue and active engagement of communities needs to minimize the delays in management to control epidemics. The aim of the study was to evaluate the relationship between sociodemographic characteristics and householders' Health-Seeking Behaviours (HSB), Dengue-Prevention Behaviours (DPB), and Community Capacities (CC) for sustained dengue prevention in Sri Lanka, a country with a high dengue endemicity.

**Methods:**

A cross-sectional analytical study was carried out in a district with the highest dengue endemicity from January to April 2019. Of the householders, 532 were chosen randomly. A pre-tested, validated, and interviewer-administered questionnaire was used to assess HSB and DPB. The HSB was assessed using three aspects, initial response for fever management, the duration of blood testing and initial response if suspected dengue. The DPB assessment was evaluated using ‘waste, outdoor water container, indoor water container, roof gutter and water storage management’. ‘Dengue Community Capacity Assessment Tool’, with 14 key items was used to assess the level of community capacity for dengue prevention. Out of the total, ≥ 50% was considered as an “adequate” HSB, DPB and CC. Multiple logistic regression was performed to control confounding effects. The results were expressed as adjusted Odds-Ratios (aOR) and 95% Confidence Intervals (CI).

**Results:**

The response rate was 93.2% (*n* = 496). Among them, 44.6% (*n* = 221) had adequate overall HSB, and 19.2% (*n* = 95) had adequate DPB. Householders who have ≤ 4 family members are 1.74 times (aOR = 1.74; 95% CI: 1.17 – 2.61) more likely to have adequate HSB and 1.85 times (aOR = 1.85; 95% CI: 1.11 – 3.09) more likely to have adequate DPB. The age group of 46 to 70 years’ individuals (aOR = 1.74; 95% CI:1.12 – 2.92), and who engaged in employment (aOR = 1.68; 95% CI: 1.05 – 2.67) were more likely to have adequate DPB than the group of 18 to 45 years and the non-employed individuals respectively. Of them, 24.6% (*n* = 122) perceived that they have adequate CC. The householders who have per-capita income < USD 50 are 1.95 times (aOR = 1.95; 95%CI:1.11 – 3.40) more likely to have adequate CC.

**Conclusion:**

The HSB, DPB and CC need to be improved to change the behaviour for sustainable dengue prevention and community capacity-building programmes need to be conducted in the Kurunegala district, Sri Lanka.

**Supplementary Information:**

The online version contains supplementary material available at 10.1186/s12889-023-15404-5.

## Introduction

The Dengue has become a major health problem in globally as well as locally. Both tropical and sub-tropical areas of the world are affected by dengue illness. According to the global epidemiological findings, approximately 40%—50% of the world’s population (2.5 to 3 billion people) which is estimated to be at risk for dengue infection in 2019. One in every 100 people contracts the dengue virus every single year, according to World Health Organization estimates. Western Pacific Region and South-East-Asia account for about 75% of the worldwide illness burden. There are currently numerous co-circulating serotypes in five countries with endemic disease [1]. The beginning of the inter-monsoonal rains causes a rise in the seasonal vector-borne disease dengue. Any action made by people who believe they have a health issue or are ill in order to locate a suitable treatment is referred to as Health Seeking Behaviour (HSB) [2]. The HSBs are critical for those who have a suspected dengue infection. Delays in seeking medical attention are strongly linked to complications leading to severe dengue. Early HSB in an individual leads to early diagnosis and timely treatment, which reduces the negative consequences of disease. Early health seeking intentions must be improved. As a result, more research on HSB is required [3, 4]. Furthermore, active community participation is required to reduce management delays and alleviate the burden [5]. In addition, individual or household behaviour, community norms, individual expectations, and provider-related characteristics all influence the HSB's decision-making process [6]. Moreover, the nature of HSB is not uniform, as it is influenced by both cognitive and non-cognitive factors. As a result, the context of HSB assessment may include cognition or awareness factors, as well as sociocultural and economic factors [7]. Given that dengue is a significant public health issue, it was necessary to address the sustainability of community-based dengue prevention and control with a specialised assessment tool [8]. In order to build a tool, test the instrument, and apply the tool to evaluate community capacity for sustainable community-based dengue prevention and control, Suwanbamrung and colleagues conducted a study in Thailand. After that, Thailand evaluated the use of a Dengue Community Capacity Assessment Tool (DCCAT) for long-term neighbourhood dengue prevention and control [9]. Therefore, there is a need for assessing behaviour for dengue control and CC in Sri Lanka too. Importantly, inter-sectoral participation, active disease surveillance, outbreak preparedness, capacity building, proper case management, proper vector control programs at multi-level, and vector control research are identified as global strategies for prevention and control of dengue by the dengue control programme [10]. When considering the perspective of the Sri Lanka on sustainable dengue prevention and control, all age groups and all 26 administrative districts have been affected with high disease transmission, becoming a leading public health problem during last two decades. Therefore, sustainable vector control is identified as key strategies in effective outbreak preparedness. Moreover, positive vector control environment has been created by proactive integrated vector management, with multisector partnership [11]. Although other health indicators are at a high standard in Sri Lanka, dengue has risen up to epidemic levels. Therefore, it is suggested to conduct continuous activities to combat the dengue epidemic situation [1]. A systematic review revealed that the behavioural change interventions are more effectively impact on the communities and sustainability of the activities [12]. After occurring of disastrous situation due to dengue outbreak during last two decades specially in 2017, there is a need to change the behavior of the community to prevent further occurrences of outbreaks. Therefore, systematically develop an intervention package with improved methodology with minimum risk of bias attempted to prevent dengue from high-risk segment of Kurunegala district, Sri Lanka. Thus, there’s a need of assessing HSB, DPB and CC in the stage of developing an intervention for dengue prevention, which enable responsible authorities to strengthen control strategies to improve dengue prevention activities in endemic area. The aim of the study was to evaluate the relationship between sociodemographic characteristics and householders' Health-Seeking Behaviours (HSB), Dengue-Prevention Behaviours (DPB), and Community Capacities (CC) for sustained dengue prevention in Sri Lanka, a country with a high dengue endemicity.

## Methods

A cross-sectional analytical study was conducted in the highest dengue endemic area in Kurunegala district, Sri Lanka during last five years. The census population in 2012 was 1,618,465 and the estimated population in 2019 was 1,719,000 [13, 14]. There are 28 preventive health sector administrative divisions called Medical Officers of Health areas (MOH) in Kurunegala district. The highest number of dengue cases have been reported in the Kurunegala MOH division during last five years. Therefore, the MOH division-Kurunegala was selected as the study setting. The study was carried out during 1^st^ of January 2019 to 10^th^ April 2019. An adult, age between 18 to 70 years in separate household was included as the study participant. Temporary residence adults who were in the area for less than six months were not included in the study since it was impossible to assess their actual behaviour and perceived level of capacity in relation to that specific area. Sample size was calculated using the critical value for the 95% confidence level set at 1.96, precision as 0.04 and expected community capacity as 30%. Therefore, the required sample size with 5% non-respondent rate was 532. The MOH division-Kurunegala has six Public Health Inspector (PHI) areas. Out of six PHI areas, one PHI area was selected randomly, and the selected area has 17 Subdivisions called ‘Grama Niladhari’ (GN) divisions. One GN division was selected randomly, and the division consists of 3.57 km^2^. A list of separate households was prepared with the available household list with the public administrative officer of the area. Out of 796 households (3689 adults), the required number of 532 individuals in the separate households were included using a random number table. An adult from one household was selected randomly if there were more than one individual in the selected household. If there were two or more families live in upstairs and downstairs or annexures in the same premises, those units were taken as separate households because the behaviour and the premises vector indices could be different from one household to another. If there was more than one adult in one household, the required person was selected randomly using the lottery method. 

An Interviewer Administered Questionnaire (IAQ) was used to collect data. The IAQ consists of four parts including ‘socio-demographic information, HSB, DPB and CC’. The IAQ was adopted from the existing literature [15–19]. Experts in the fields of Public health and General Medicine were invited for a consultative meeting for assessing the face, content, and consensual validity of the questionnaire. With the expert opinion and existing literatures, the marking scheme was developed. The HSB were enquired through close ended questions. There were pre-coded options for each item of the questioner. If any of the mentioned options were not relevant, respondents were asked to specify their action for each question. The HSB was assessed using three aspects including, initial response for the fever management, duration for blood testing and initial response if suspected dengue. Total part was given 30 marks according to the marking scheme and converted into percentage (Range 0 – 100%). The overall HSB was categorized in to two groups as “adequate” and “inadequate”. The “adequate behaviour” was described as taking ≥ 50% of total score. The DPB assessment was consisted of five parts. It was evaluated using ‘waste management (25 marks), outdoor water container management (10 marks), indoor water container management (10 marks), roof gutter management (10 marks) and water storage management (10 marks) which were observed by the interviewers. Waste management was assessed by 10 broad areas covering the 3R concept (Reduce, Reuse and Re-cycling). Total part of the DPB was given 65 marks and the percentage was taken for the overall prevention behaviour. The overall DPB was categorized in to two groups as “adequate” and inadequate”. The “adequate behaviour” was described as taking ≥ 50% of total score. Modified ‘Dengue Community Capacity Assessment Tool’ (DCCAT) was used to assess the level of CC on dengue prevention. It produced the best fit regarding content validity (CVI = 0.90), construct validity (Commutative percent of variance = 57.58), and Cronbach's alpha coefficient (0.98). It has 14 key items including ‘critical situation management, personal leadership, health care provider capacity, needs assessment, senses of community, leader group networking, communication of dengue information, community leadership, religious capacity, leader group and community networking, resource mobilization, dengue working group, community participation, and continuing activities’ [18]. It was measured by five-point Likert scale which was categorized in to “Very High (5)”, “High (4)”, “Moderate (3)”, “Low (2)”, and “Very low (1)” capacity groups. Zero marks were given for the “Not sure” answers. The numbers and the percentages for each item were described according to the categories of Likert scale (5–4-3–2-1–0). The scale was given zero to 70 marks for the tool and percentage was taken for each tool. Out of total, ≥ 50% was considered as an “adequate community capacity”. The questionnaire was initially prepared in English language and then translated to local languages using forward–backward translation methodology. Data was collected by four trained data collectors. Five training sessions were conducted to train all the components of the IAQ, and four mock interviews were conducted to assess the skills of communication and data gathering. The IAQ was pre-tested among 25 householders in another area. Household observations were conducted by the trained data collectors to assess the behaviours in order to fill the instrument. Data were manually checked and cleaned before entering into the data base. Coding of each variable was done. Data analysis was conducted using the Statistical Package for the Social Sciences (SPSS)-20^th^ version. Socio-demographic characteristics, percentage of adequate DPB and adequate CC were described in relation to the socio-demographic characteristic of the study population. Standard of Living Index (SOLI) (Refer Additional file 4) was calculated using the Modified SOLI by Munasinghe, 2002 to classify the individual’s social status which was based on demographic and Health survey format worldwide was taken to assess the SOLI of the participants [15]. Chi squared test was applied to assess the association between socio-demographic characteristics, DPB and CC. Multiple logistic regression was performed to control the confounding effects. The results were expressed as adjusted Odds Ratios (aOR) and 95% Confidence Intervals (CI).

## Results

Out of 532 invited, 496 participated giving a response rate of 93.2%. The mean age was 46.4 years (SD = 12.6) with the range of 18 to 70 years. The highest proportion of the participants was age between 41 to 50 years (*n* = 135; 27.2%). The majority of the participants were female (*n* = 259; 52.2%), Sinhalese (*n* = 476; 96.0%), married (*n* = 176; 92.1%), had passed General Certificate Exam Ordinary Level (G.C.E. O/L) (*n* = 168, 33.9%), and engaged in paying employments (*n* = 274; 55.2%). The median duration of the living of the area was 26.5 years (IQR 15 – 26.5 years). The median number of family members was four. The median per capita monthly income was USD 50 [IQR = 43—56) [SLR 175 = USD in 2019].

### Heath Seeking Behaviour (HSB)

The majority of the participants had inadequate HSB in relation to all aspects. The median score of the HSB was 46.7 (IQR = 33.3 to 46.7) with the range from 20 to 100%. The majority had inadequate behavioural habits when having fever (*n* = 254; 51.2%), Blood testing following fever (*n* = 420; 84.7%) and when suspecting dengue (*n* = 274; 55.2%) (Table 1). Out of the study population, 44.6% (*n* = 221) had adequate overall HSB. Out of the demographic characteristics of the study group, there were statistically significant (*p* < 0.5) associations between the adequate HSB with the number of family members, and per capita monthly income (Table 2). Multiple logistic regression showed that individuals who have four or less family members are 1.74 times (95% CI: 1.17 – 2.61) more likely to have adequate HSB than the individuals who have more than four family members (Refer Additional file 1_Table S 1).

**Table 1:** Distribution of the Study Population by Three Aspects of Health Seeking Behaviours

**Aspects of Health Seeking Behaviours**	**Category**	**n (%)**
**1) Fever management**	Adequate	242 (48.8)
	Inadequate	254 (51.2)
**2) Blood testing**	Adequate	76 (15.3)
	Inadequate	420 (84.7)
**3) Suspect dengue**	Adequate	222 (44.8)
	Inadequate	274 (55.2)

**Table 2:** Association of Socio-demographic Characteristics of the Study Population and their Health Seeking Behaviour

**Variable**	**Health Seeking Behaviour**		**Test of significant**
	**Adequate**	**Inadequate**	
	**n (%)**	**n (%)**	
**Age category (years)**			
18 to 45 years	95 (41.7)	133 (58.3)	χ 2 (*d.f*. = 1, *N* = 496) = 1.42
46 to 70 years	126 (47.0)	142 (53.0)	*p* = 0.23
**Gender**			
Female	111 (42.9)	148 (57.1)	χ 2 (*d.f*. = 1, *N* = 496) = 0.63
Male	110 (46.4)	127(53.6)	*p* = 0.43
**Nationality**			
Non-Sinhalese	9 (45.0)	11 (55.0)	χ 2 (d.f. = 1, *N* = 496) = 0.002
Sinhalese	212 (44.5)	264 (55.5)	*p* = 0.97
**Marital status**			
Other	26 (49.1)	27 (50.9)	χ 2 (*d.f*. = 1, *N* = 496) = 0.49
Married	195 (44.0)	248 (56.0)	*p* = 0.49
**Level of education**			
Above O/L	82(41.2)	117 (58.8)	χ 2 (*d.f*. = 1, *N* = 496) = 1.51
O/L or below	139(46.8)	158 (53.2)	*p* = 0.22
**Occupation**			
Employed	129 (47.1)	145 (52.9)	χ 2 (*d.f*. = 1, *N* = 496) = 1.58
Non-employed	92 (41.4)	130 (58.6)	*p* = 0.21
**Duration of living**			
36 to 70 years	91 (46.9)	103 (53.1)	χ 2 (*d.f.* = 1, *N* = 496) = 0.71
1 to 35 years	130 (43.0)	172 (57.0)	*p* = 0.34
**Family members per family**			
One to four	162 (50.0)	162 (50.0)	χ 2 (d.f. = 1, *N* = 496) = 11.23
Five to Eight	59 (34.3)	113 (65.7)	***p*** = 0.001*
**Household monthly income**			
Less than USD 200	113 (42.3)	154 (57.7)	χ 2 (*d.f.* = 1, *N* = 496) = 1.17
More than USD 200	108 (47.2)	121 (52.8)	*p* = 0.28
**Per capita monthly income**			
USD 50 or less	97 (38.6)	154 (61.4)	χ 2 (*d.f.* = 1, *N* = 496) = 7.18
More than USD 50 *	124 (50.6)	121 (49.4)	***p*** = 0.007^*^
**Standard of Living Index (SOLI)**			
6 or less	103 (39.8)	156 (60.2)	χ 2 (*d.f.* = 1, *N* = 496) = 5.03
More than 6	118 (49.8)	119 (50.2)	***p*** = 0.025^*^

^*^The χ 2 is significant at the 0.05 level

### Dengue prevention behaviours

The majority of the participants did not have adequate breeding places management (*n* = 401; 80.8%) and adequate waste management practices at the households (*n* = 389; 78.4%). Among the available participants, 233 (63.5%) had inadequate behaviours on water container management, 185 (62.7%) did not have adequate behaviours on roof gutter management, and 197 (57.6%) did not have adequate behaviour on indoor container management (Table 3). The mean overall DPB of the householders was 38.5 (SD = 13.4) with the range from 12 to 83. Only 19.2% (*n* = 95) had adequate DPB. There were statistically significant (*p* < 0.5) associations between the DPB with age, occupation, and number of family members (Table 4). Multiple logistic regression showed that the age group of 46 to 70 years are more likely to have adequate DPB than the group of 18 to 45 years’ age category with an aOR of 1.74 (95% CI: 1.12 – 2.92). The individuals who engaged in employments are more likely to have adequate DPB than the non-employed individuals with an aOR of 1.68 (95% CI: 1.05 – 2.67). The families who have less than four members are more likely to have adequate DPB than the families with more than four members with an aOR of 1.85 (95% CI: 1.11 – 3.09) (Refer Additional file 1_Table S 2).

**Table 3:** Distribution of the Study Population by Five Aspects of Dengue Prevention Behaviours

**Dengue Prevention Behaviours Category**		**n (%)**
**1) Outdoor Breeding Places Management**	Adequate	93 (18.8)
	Inadequate	403 (81.2)
**2) Water storage management**	Adequate	134 (36.5)
	Inadequate	233 (63.5)
	Not available	129 (26.0)
**3) Roof gutter management**	Adequate	110 (37.3)
	Inadequate	185 (62.7)
	Not available	201(40.5)
**4) Indoor Breeding Places Management**	Adequate	145 (42.4)
	Inadequate	197 (57.6)
	Not available	154 (31.0)
**5) Household Waste Management**	Adequate	107 (21.6)
	Inadequate	389 (78.4)

**Table 4:** Association of Socio-demographic Characteristics of the Study Population and their Dengue Prevention Behaviour

**Variable**	**Dengue Prevention Behaviour**		**Test of significant**
	**Adequate**	**Inadequate**	**(χ 2; ** ***p-*** **value)**
	**n (%)**	**n (%)**	
**Age category (years)**			
18 to 45 years	32 (14.0)	196 (86.0)	**χ 2 (** ***d.f*** **. = 1, ** ***N*** ** = 496) = 7.14**
46 to 70 years	63 (23.5)	205 (76.5)	***p*** = 0 .008*
**Gender**			
Male	48 (20.3)	205 (76.5)	χ 2 (*d.f*. = 1, *N* = 496) = 0.36
Female	47 (18.1)	212 (81.9)	*p* = 0.55
**Nationality**			
Non-Sinhalese	01 (5.0)	19 (95.0)	χ 2 (*d.f*. = 1, *N* = 496) = 2.70
Sinhalese	94 (19.7)	382 (80.3)	*p* = 0.10
**Marital status**			
Other	11 (20.8)	42 (79.2)	χ 2 (*d.f*. = 1, *N* = 496) = 0.*10*
Married	84 (19.0)	359 (81.0)	*p* = 0.75
**Level of education**			
Above O/L	40 (20.1)	159 (79.9)	χ 2 (*d.f*. = 1, *N* = 496) = 0.19
O/L or below	55 (18.5)	242 (81.5)	*p* = 0.66
**Occupation**			
Employed	62 (22.6)	212 (77.4)	χ 2 (d.f. = 1, *N* = 496) = 4.77
Non-employed	33 (14.9)	189 (85.1)	***p*** = 0.029*
**Duration of living**			
36 to 70 years	41 (21.1)	153 (78.9)	χ 2 (*d.f.* = 1, *N* = 496) = 0.81
1 to 35 years	54 (17.9)	248 (82.1)	*p* = 0.37
**Family members per family**			
One to four	72 (22.2)	252 (77.8)	χ 2 (*d.f.* = 1, *N* = 496) = 5.68
Five to Eight	23 (13.4)	149 (86.6)	***p*** = 0.017^*^
**Household monthly income**			
Less than USD 200	56 (21.0)	211 (79.0)	χ 2 (*d.f.* = 1, *N* = 496) = 1.24
More than USD 200	39 (17.0)	190 (83.0)	*p* = 0.27
**Per capita monthly income**			
USD 50 or less	50 (19.9)	201(80.1)	χ 2 (*d.f.* = 1, *N* = 496) = 0.19
More than USD 50	45 (18.4)	200 (81.6)	*p* = 0.66

*95% CI* 95% Confidence Interval, *OR* Odds Ratio

^*^ The χ 2 is significant at the 0.05 level

SLR 175 = USD 1 in 2019

### Community capacity

The percentages of each domain of CC assessment are presented by Table 5. The mean score of the overall CC was 59.81 (SD = 13.6) with the rage from 36 to 100%. The majority (*n* = 374; 75.4%) of the study population perceived that they have inadequate level of capacity for dengue management. Out of the demographic characteristics, there were statistically significant (*p* < 0.5) associations between the adequate CC with the number of per capita monthly income (Table 6). Multiple logistic regression revealed that the individuals who have per capita income less than USD 50 are 1.95 times (aOR) (95% CI: 1.11—3.40) more likely to have adequate CC than the individuals who have the per capita income of more than USD 50 per month (*p* = 0.02). Age categories, sex, nationality, marital status, occupation, duration of living in the same area and household monthly income were not significantly associated with adequate CC (Table 6).

**Table 5:** Community Capacity for the Prevention of Dengue Among the Study Participants

**Domains**	**Very** **High** **n** **(%)**	**High** **n** **(%)**	**Moderate** **n** **(%)**	**Low** **n** **(%)**	**Very low** **n** **(%)**	**Not sure** **n** **(%)**	**Domains**
**1**	**Critical situation management ability of the community for dengue management**	28 (5.6%)	68 (13.7%)	**263** **(53%)**	129 (26.0%)	5 (1%)	3 (0.6%)
**2**	**Personal leadership ability of the community**	32 (6.5%)	47 (9.5%)	123 (24.8%)	**282** **(56.9%)**	12 (2.4%)	0 (0%)
**3**	**Health care provider’s capacity of the area**	63 (12.7%)	151 (30.4%)	**251** **(50.6%)**	22 (4.4%)	7 (1.4%)	2 (0.4%)
**4**	**Need assessment for dengue prevention activities of the area**	40 (8.1%)	117 (23.6%)	**262** **(52.8%)**	66 (13.3%)	5 (1.0%)	6 (1.2%)
**5**	**Sense of the community as dengue is a problem of the area**	62 (12.5%)	84 (16.9%)	**169** **(34.1%)**	90 (18.1%)	78 (15.7%)	13 (2.6%)
**6**	**Leader group networking of the area**	35 (7.1%)	132 (26.6%)	**187** **(37.7%)**	115 (23.2%)	13 (2.6%)	14 (2.8%)
**7**	**Communication of dengue information of the area**	30 (6%)	121 (24.4%)	**222** **(44.8%)**	95 (19.2%)	11 (2.2%)	17 (3.4%)
**8**	**Community leadership on Dengue prevention of the area**	41 (8.3%)	137 (27.6%)	**189** **(38.1%)**	109 (22.0%)	9 (1.8%)	11 (2.2%)
**9**	**Religious leader capacity of the area**	54 (10.9%)	**204** **(41.1%)**	194 (39.1%)	29 (5.8%)	7 (1.4%)	8 (1.6%)
**10**	**Leader group and community networking of the area**	38 (7.7%)	126 (25.4%)	**216** **(43.5%)**	99 (20.0%)	3 (0.6%)	14 (2.8%)
**11**	**Resource mobilization for dengue prevention activities of the area**	38 (7.7%)	122 (24.6%)	**228** **(46.0%)**	98 (19.8%)	3 (0.6%)	7 (1.4%)
**12**	**Dengue working group of your area**	74 (14.9%)	**168** **(33.9%)**	152 (30.6%)	88 (17.7%)	5 (1.0%)	9 (1.8%)
**13**	**Community participation of the area**	55 (11.1%)	150 (30.2%)	**196** **(39.5%)**	82 (16.5%)	2 (0.4%)	11 (2.2%)
**14**	**Continuity of activities on dengue prevention of the area**	62 (12.5%)	88 (17.7%)	**183** **(36.9%)**	142 (28.6%)	5 (1.0%)	16 (3.2%)

**Table 6:** Association of Demographic Characteristics of the Study Population and their Community Capacity

**Variable**	**Community capacity**		**Test of significant**
	**Adequate**	**Inadequate**	
	**n (%)**	**n (%)**	
**Age category (years)**			
18 to 45 years	173 (75.9)	55 (24.1)	χ 2 (*d.f*. = 1, *N* = 496) = 0.03
46 to 70 years	205 (76.5)	63 (23.5)	*p* = 0.87
**Gender**			
Male	185 (78.1)	52 (21.9)	χ 2 (*d.f*. = 1, *N* = 496) = 0.86
Female	193 (74.5)	66 (25.5)	*p* = 0.36
**Nationality**			
Non-Sinhalese	13 (65.0)	7 (35.0)	χ 2 (d.f. = 1, *N* = 496) = 1.44
Sinhalese	365 (76.7)	111 (23.3)	*p* = 0.23
**Marital status**			
Other	42 (79.2)	11 (20.8)	χ 2 (d.f. = 1, *N* = 496) = 0.30
Married	336 (75.8)	107 (24.2)	*p* = 0*.58*
**Level of education**			
Above O/L	154 (77.4)	107 (24.2)	χ 2 (d.f. = 1, *N* = 496) = 0.25
O/L or below	224 (75.4)	45 (22.6)	*p* = 0.61
**Occupation**			
Employed	206 (75.2)	68 (24.8)	χ 2 (d.f. = 1, *N* = 496) = 0.36
Non-employed	172 (77.5)	50 (22.5)	*p* = 0*.*55
**Duration of living**			
36 to 70 years	152 (78.4)	42 (21.6)	χ 2 (*d.f.* = 1, *N* = 496) = 0.81
1 to 35 years	226 (74.8)	76 (25.2)	*p* = 0.36
**Family members per family**			
One to four	243 (75.0)	81 (25.0)	χ 2 (*d.f.* = 1, *N* = 496) = 0.75
Five to Eight	135 (78.5)	37 (21.5)	*p* = 0.38
**Household monthly income**			
Less than USD 200	204 (81.3)	47 (18.7)	χ 2 (*d.f.* = 1, *N* = 496) = 0.91
More than USD 200	174 (71.0)	71 (29.0)	*p* = 0.34
**Per capita monthly income**			
USD 50 or less	204 (81.3)	47 (18.7)	χ 2 (d.f. = 1, *N* = 496) = 7.19
More than USD 50	174 (71.0)	71 (29.0)	***p*** = 0.007*

^*^ The χ 2 is significant at the 0.05 level

There is no statistically significant association with three categories of HSB and overall HSB, CC with DPB (Refer Additional file 1_ Table S 3 & S 4).

## Discussion

Households who have four or less family members are 1.74 times more likely to have adequate HSB and 1.85 times higher likelihood of adequate DPB, according to the study. Only one-fourth of the study population perceived that they have adequate capacity for dengue management. Delays in seeking medical attention are strongly linked to severe dengue complications. It is critical to reinforce patients' intentions to seek early health care. According to the household survey, only 43.2 percent of early HSB in Myanmar were from the public sector, and 46.1 percent began with self-medication. Myanmar was able to reduce the effects on its population due to timely implementation of relevant HSB improvements [20]. It is necessary to conduct awareness campaigns among the local population in order to reduce the spread and societal burden. According to a 2013–2014 annual national survey in Venezuela, the majority of people had a positive perception of the risk of dengue, which improved HSB. The factors associated with early, adequate HSB were the ability to diagnose a dengue case at home and being an adult. However, prior dengue infection has a negative correlation with early HSB [4]. More than half of the world's population lives in areas where dengue transmission is possible. However, policymakers failed to prioritise it when allocating resources and planning interventions [21]. There is a lack of early treatment seeking, diagnosis, and timely treatment, all of which influence prognosis without having negative consequences [4]. Furthermore, HSB are critical for people who have a dengue infection. The suspected patient's intention to seek medical care early in the disease attack needs to be improved. More research is needed to investigate local health beliefs and practises, factors influencing HSB, and dengue fever access to care in order to identify challenges and opportunities in diagnostics and treatment [3]. In the current study, HSB was considered one of the key variables, and the findings were used to develop an intervention to change community behaviour for long-term dengue prevention. In contrast to the current study, a cross-sectional study in Myanmar found that HSB for suspected Dengue Fever (DF) are ineffective after comparing two villages with and without DF cases [20]. In the current study, the majority of participants received treatment from public sector health care institutions if they had fever and suspected dengue. In contrast to the current study, the majority of participants in Myanmar did not seek treatment from public health facilities when they developed a fever. However, similar to the current study findings, their perceived awareness of DF significantly influenced their HSB [21]. Similar findings to Liu's study were discovered in Venezuela [4] and Malaysia [22]. Less family-oriented households practise good HSB and dengue prevention. If there were fewer members, they would have paid more attention to preventive measures instead of waiting for others.

Given that dengue is a significant public health issue, it was necessary to address the sustainability of community-based dengue prevention and control with a specialised assessment tool [8]. Suwanbamrung and colleagues conducted research in Thailand with the goal of developing, testing, and implementing a measure to assess community capacity for long-term community-based dengue prevention and control. The procedure resulted in the creation of the DCCAT, and the evaluation was carried out through pilot testing. Following that, in Thailand, the effectiveness of a DCCAT for long-term neighbourhood-based dengue prevention and control was evaluated, incorporating both qualitative and quantitative methodology to create the final tool known as the DCCAT for leaders and non-leaders [9, 17, 23]. After being modified by a field-specific expert panel, the community's capacity can be evaluated using this verified tool. Therefore, for the study purpose, the tool was utilized. The knowledge on dengue is not sufficient to produce a behavior change [24]. However, changes in knowledge or attitudes are sometimes an important initial step before a behaviour is changed. If the behaviuor is changed by improving HSB and CC which lead to reduce the risk of the disease of interest. Furthermore, the community members need to have positive attitudes to carry out recommended behaviours [25, 26]. Being this study is a part of development of an intervention for dengue prevention through behavioural change, when developing capacity development programmes, all the aspects need to be considered.

## Conclusion and recommendations

HSB, DPB and CC need to be improved to change the behaviour for sustainable dengue prevention. Multi-stakeholder participation can ensure community participation in any setting. We have a well-established preventive sector, particularly in an Asian country like Sri Lanka. As a result, community empowerment programmes are simple to implement in the local context. To prevent and control dengue, community capacity assessments and community empowerment programmes must be conducted on a regular basis, and sustainability must be ensured through multi-stakeholder participation. The current study was conducted with minimal disruption to the participants' routine activities as consented householders. Capacity-building programmes could be implemented by leveraging existing public-sector infrastructure and involving multiple stakeholders. After assessing baseline capacity assessments surveys, it is critical to estimate the public health impact of dengue interventions in order to empower policymakers to deploy the most effective and efficient interventions in resource-limited settings. To improve behaviour change, empirical evidence of the effectiveness of a community-wide dengue vector control programme could be planned. By providing baseline data on the areas of Sri Lanka with the highest dengue endemicity, the findings of this study enabled the development of community capacity building programmes.

### Limitation

The study limited to the highly endemic MOH area out of 28 MOH areas in Kurunegala district, Sri Lanka which partially reflects the situation in the country. However, Kurunegala district is one of the major districts out of 26 administrative districts in Sri Lanka. It is one of the highest dengue endemic four districts in Sri Lanka, with the estimated population of 1,719,000 in 2019 [12, 13].

## Supplementary Information

**Additional file 1: Table S1.** Determinants of the Adequate Health Seeking Behaviour. **Table S2.** Determinants of the Adequate Dengue Prevention Behaviour. **Table S3.** Association between Health Seeking Behaviour and Dengue Prevention Behaviours. **Table S4.** Association Between Overall Health Seeking Behaviour, Community Capacity and Dengue Prevention Behaviours.

**Additional file 2.**

**Additional file 3.**

**Additional file 4.**

## Data Availability

The dataset is available from the main corresponding author (principal investigator) on reasonable request.

## Abbreviations

CC: Community Capacity
DF: Dengue Fever
DPB: Dengue-Prevention Behaviours
DCCAT: Dengue Community Capacity Assessment Tool
GCE O/L: General Certificate Examination Ordinary Level
HSB: Health-Seeking Behaviour
GN: ‘Grama Niladhari’
IQR: Inter Quartile Range
IAQ: Interviewer Administered Questionnaire
OR: Odds Ratio
MOH: Medical Officers of Health
PHI: Public Health Inspector
SD: Standard Deviation
SEA: South-East Asia
SPSS: Statistical Package for the Social Sciences
SOLI: Standard of Living Index
SLR 175 = USD 1 in 2019: Sri Lanka Rupees
95% CI: Confidence Interval
