# Supplementary material for: Health seeking behaviours, dengue prevention behaviours and community capacity for sustainable dengue prevention in a highly dengue endemic area, Sri Lanka
Source: BMC Public Health. 2023 Mar 16;23:507. doi: 10.1186/s12889-023-15404-5 (PMC10022255; doi:10.1186/s12889-023-15404-5)
Supplement: Supplementary file 1 — Additional file 1: Table S1. Determinants of the Adequate Health Seeking Behaviour. Table S2. Determinants of the Adequate Dengue Prevention Behaviour. Table S3. Association between Health Seeking Behaviour and Dengue Prevention Behaviours. Table S4. Association Between Overall Health Seeking Behaviour, Community Capacity and Dengue Prevention Behaviours. [file 12889_2023_15404_MOESM1_ESM.docx]

**Additional file 1**

**Table S 1. Determinants of the Adequate Health Seeking Behaviour**

| **Variable** | **β** | **SE** | **aOR** | **95%CI** | **p value** |
| --- | --- | --- | --- | --- | --- |
| **Family members (< 4)** | 0.56 | 0.21 | 1.74 | 1.17 – 2.61 | **0. 007**^*^ |
| **Per capita monthly income (<USD 50)** | 0.36 | 0.24 | 1.43 | 0.89 – 2.30 | 0.15 |
| **SOLI (<6)** | 0.44 | 0.23 | 1.36 | 0.89 – 2.11 | 0.31 |
| **Constant** | 0.21 | 0.17 | 1.24 |  |  |

β – Regression Coefficient, SE – Standard Error, aOR – Adjusted Odds Ratio, CI – Confidence interval

**Table S 2. Determinants of the Adequate Dengue Prevention Behaviour**

| **Variable** | **β** | **SE** | **aOR** | **95%CI** | ***p* value** |
| --- | --- | --- | --- | --- | --- |
| Age (18 to 45 years) | 0.56 | 0.24 | 1.74 | 1.12 – 2.92 | **0.02*** |
| Occupation (employed) | 0.44 | 0.24 | 1.68 | 1.05 – 2.67 | **0.07*** |
| Family members (<4) | 0.55 | 0.26 | 1.85 | 1.11 – 3.09 | **0.04*** |
| Constant | 1.30 | 0.23 | 3.68 |  | 0.00 |

β – Regression Coefficient, SE – Standard Error, aOR – Adjusted Odds Ratio, CI – 95% confidence interval

**Table S 3**

**Association between Health Seeking Behaviour and Dengue Prevention Behaviours**

|  | | **Dengue Prevention Behaviours** | | **Test of significant** |
| --- | --- | --- | --- | --- |
|  |  | **Adequate** | **Inadequate** |  |
|  |  | **n (%)** | **n (%)** |  |
| 1. **Fever** | Adequate | 47 (19.4) | 195 (80.6) | χ 2 (d.f.= 1) = 0.022 |
|  | Inadequate | 48 (18.9) | 206 (81.1) | *p*=0.88 |
| 1. **Blood testing** | Adequate | 15 (19.7) | 61 (80.3) | χ 2 (d.f.= 1) = 0.020 |
|  | Inadequate | 80 (19.0) | 340 (81.0) | *p*=0.88 |
| 1. **Dengue** | Adequate | 43 (19.4) | 179 (80.6) | χ 2 (d.f.= 1) = 0.011 |
|  | Inadequate | 52 (19.0) | 222 (81.0) | *p*=0.912 |

**Table S4**

**Association Between Overall Health Seeking Behaviour, Community Capacity and Dengue Prevention Behaviours**

|  | | **Dengue Prevention Behaviours** | | **Test of significant** |
| --- | --- | --- | --- | --- |
|  |  | **Adequate** | **Inadequate** |  |
|  |  | **n (%)** | **n (%)** |  |
| **Overall HSB** | Adequate | 43 (45.3) | 178 (44.4) | χ 2 (d.f.= 1) = 0.02 |
|  | Inadequate | 52 (54.7) | 223 (55.6) | *p*=0.88 |
| **Community Capacity** | Inadequate | 68 (71.6) | 306 (76.3) | *χ 2 (d.f.= 2) = 3.39* |
|  | Adequate | 27 (28.4) | 95 (23.7) | *p*=0.18 |

* The χ 2 is significant at the 0.05 level: HSB=Health Seeking Behaviour
