# Supplementary material for: Health seeking behaviours, dengue prevention behaviours and community capacity for sustainable dengue prevention in a highly dengue endemic area, Sri Lanka
Source: BMC Public Health. 2023 Mar 16;23:507. doi: 10.1186/s12889-023-15404-5 (PMC10022255; doi:10.1186/s12889-023-15404-5)
Supplement: Supplementary file 2 — Additional file 2. [file 12889_2023_15404_MOESM2_ESM.docx]

|  | Only for the purpose of research team |
| --- | --- |
| Name of the interviewer: |  |
| “Grama Niladhari” division: |  |

Part 01: Socio-demographic factors

|  |
| --- |

1. What is your Age?

| Male | Female |
| --- | --- |

1. What is your sex?

1. What is your ethnicity?

| 1. | Sinhala |  |
| --- | --- | --- |
| 2. | Tamil |  |
| 3. | Muslim |  |
|  | Other (please specify) |  |

1. What is your marital status?

| 1. | Married |  |
| --- | --- | --- |
| 2. | Never married |  |
| 3. | Divorced |  |
| 4. | Widowed |  |
|  | Other (please specify) |  |

1. What is your highest level of education?

| 1. | Degree |  |
| --- | --- | --- |
| 2. | Diploma passed |  |
| 3. | GCE (A/L) passed |  |
| 4. | GCE (O/L) passed |  |
| 5. | Grade 11 or less |  |
| 6. | Grade 5 or less |  |
| 7. | No schooling at all |  |
| 8. | Other (please specify)  ………………………………………….. |  |

|  |
| --- |

1. What is the main occupation of the household owner?

| Years |
| --- |

1. What is the duration of residence in this area?

|  |
| --- |

1. How many occupants living in this house?

1. What is your average monthly income?

1. Does your household have?

| 1. | Electricity |  |
| --- | --- | --- |
| 2. | Radio |  |
| 3. | TV |  |
| 4. | Mobile phone/ land phone / both (please specify) |  |
| 5. | Refrigerator |  |
| 6. | Generator/ battery/ Solar power |  |
| 7. | A bicycle |  |
| 8. | A motor bike |  |
| 9. | Three wheel |  |
| 10. | A car/ Van/ Bus/ Other vehicle ( please specify) |  |

Part 01: Health Seeking Behaviour

1. If you think you or someone in your family has fever, what would you do first? (only one answer)

| Take drug from the pharmacy | | | | |  |
| --- | --- | --- | --- | --- | --- |
|  | Go to the private provider (without considering the qualification of doctors) | | |  |  |
| Go to the | | public sector | (Western/Ayurwedhic) healthcare provider | |  |
| Engage in religious activities, till symptoms disappear | | | | |  |
| Stay at home till the fever goes off | | | | |  |
| Other (please specify)…………………………………………………………………………… | | | | | |

1. If you or (a family member) get fever (temperature more than 99c), how many days you wait to check blood count after symptoms start? (only one answer)

|  | Time |  |  | NS1 |  |  | FBC |  |
| --- | --- | --- | --- | --- | --- | --- | --- | --- |
| Same day (Day 0) | | |  | |  |  | | |
| Day 01 | | |  | |  |  | | |
| Day 02 | | |  | |  |  | | |
| Day 03 | | |  | |  |  | | |
| Do not allocate time to check blood | | |  | |  |  | | |
| Other (please specify) | | |  | |  | | | |

1. If you suspect you or someone in your family has dengue, what would you do first? (only one answer)

| Take drug from the pharmacy | | | | |  |
| --- | --- | --- | --- | --- | --- |
|  | Go to the private provider (without considering the qualification of doctors) | | |  |  |
| Go to the | | public sector | (Western/Ayurwedhic) healthcare provider | |  |
| Engage in religious activities, till symptoms disappear | | | | |  |
| Stay at home till the fever goes off | | | | |  |
| Other (please specify)…………………………………………………………………………… | | | | | |

Part 02: Dengue Prevention Behaviours (Record observation and ask)

1. Outdoor: What did they do for the discarded empty containers which breeds mosquitos?

| Through them in to a rubbish heap in the backyard | Yes | No |
| --- | --- | --- |
| Heap them at some other place on the roadside | Yes | No |
| Buried in the premises | Yes | No |
| Handed over suitable items for recycling | Yes | No |
| Handed over to the garbage collecting vehicle of the “Pradeshiya Sabha”/other | Yes | No |

| 2. | Indoor: Did they have any water collection at indoor like refrigerator trays, flower vas etc.? |
| --- | --- |

| Yes | No |
| --- | --- |

If yes, how did they maintain those?

|  | Empty, clean and scrub weekly and those are cleaned | | |  | |  |
| --- | --- | --- | --- | --- | --- | --- |
|  | Empty, clean and scrub weekly; but those are not cleaned | | | |  |  |
|  | Empty weekly and those are not cleaned | |  | | |  |
|  | Empty whenever it is overfilled and fill |  | | | |  |
|  | Don’t pay any special attention for that |  | | | |  |

3. Water Storage management: Did they store water in a tank or barrel in their premises?

If yes, what do they do to keep water clean in their water tank/ barrel?

| Do not have time to pay any special attention |  |
| --- | --- |
| Keep it closed with a lid; but not clean |  |
| Empty, clean, scrub and refill fortnightly |  |
| Empty, clean, scrub and refill weekly |  |
| Clean, scrub whenever it becomes empty and those are well maintained |  |

| Yes | No |
| --- | --- |

1. Are there rain gutters in the household?

If yes, how did they maintain rain gutters?

| Flush out whenever there is stagnant water | Yes | No |
| --- | --- | --- |
| Cut trees close to the gutters to ensure proper drainage | Yes | No |
| Replace damaged/bend gutters to ensure proper drainage | Yes | No |
| Flush out and scrub whenever there is stagnant water | Yes | No |
| Don’t have time to pay any special attention | Yes | No |

Other (please specify)......…………………………………………………………………………………

3. Waste Management According to 3R concept: How did they manage household waste

(Record observation and ask from the participant)

|  | | | Waste Management Behaviour | | |  | | | | | | |  |  | | Observation | | | |  | |
| --- | --- | --- | --- | --- | --- | --- | --- | --- | --- | --- | --- | --- | --- | --- | --- | --- | --- | --- | --- | --- | --- |
|  |  |  |  | | |  |  |  |  |  |  |  |  |  |  |  | | | |  |  |
| 1. |  | Reduce the use of polyene and plastic as a habit | | | | | | | |  | | |  | Yes | | | | No | | | |
|  |  |  | | | | | | | |  |  |  |  |  |  |  |  |  |  |  |  |
| 2. | Re-use polythene and plastics using | | | | | | | flower vas creation etc. | | | | |  | Yes | | | | No | | | |
|  |  |  |  |  |  |  |  |  | | | | |  |  |  |  |  |  |  |  |  |
| 3. | Burn polythene and plastics | | | | inside the house | | | |  | | | |  | Yes | | | | No | | | |
|  |  |  |  |  |  | | | |  |  |  |  |  |  |  |  |  |  |  |  |  |
| 4. | Burn polythene and plastics | | | | outside the house | | | | | | |  |  | Yes | | | | No | | | |
|  |  |  |  |  |  | | | | | | |  |  |  |  |  |  |  |  |  |  |
| 5. | Discarded the batteries to the environment | | | | | | | | | | | |  |  | Available | |  |  | Not available | |  |
|  |  |  |  |  |  |  |  |  |  |  |  |  |  |  |  | |  |  |  | |  |
| 6. | Handover to | | | “Pradeshiya Sabha | | | ” vehicle /Other collecting | | | | | | places | Yes | | | | No | | | |
|  |  |  |  |  | | |  |  |  |  |  |  |  |  |  |  |  |  |  |  |  |
| 7. | Recycling the non-degradable waste | | | | | | | like Tin, Plastic | | |  | |  | Yes | | | | No | | | |
|  |  |  |  |  |  |  |  |  | | |  |  |  |  |  |  |  |  |  |  |  |
| 8. | Composting the biological waste | | | | | | (any method) | | | | |  |  |  | Available | |  |  | Not available | |  |
|  |  |  |  |  |  |  |  | | | | |  |  |  |  | |  |  |  | |  |
| 9. |  | Use compost for home gardening | | | | |  | | | | | |  |  | Available | |  |  | Not available | |  |
|  |  |  | | | | |  |  |  |  |  |  |  |  |  | |  |  |  | |  |
| 10. |  | Segregate the daily waste in to separate bins | | | | | | |  | | | |  |  | Available | |  |  | Not available | |  |
|  |  |  | | | | | | |  |  |  |  |  |  |  | |  |  |  | |  |

Any other: please specify

………………………………………………………………………………………………………………………………………………………………………………………………………………………………………………………………………………………………………………………………………………………………………………………………………………………………………………………………………………………………………………………………………………………………………………………………………………………………………………………………………………………………

………………………………………………………………………………………………………………

(Thank you for participating this survey)
