# Supplementary material for: Health seeking behaviours, dengue prevention behaviours and community capacity for sustainable dengue prevention in a highly dengue endemic area, Sri Lanka
Source: BMC Public Health. 2023 Mar 16;23:507. doi: 10.1186/s12889-023-15404-5 (PMC10022255; doi:10.1186/s12889-023-15404-5)
Supplement: Supplementary file 3 — Additional file 3. [file 12889_2023_15404_MOESM3_ESM.docx]

|  | **Domains** | **Very**  **High**  **5** | **High**  **4** | **Moderate**  **3** | **Low**  **2** | **Very low**  **1** | **Not sure**  **0** |
| --- | --- | --- | --- | --- | --- | --- | --- |
| **1** | **Critical situation management ability of your community for dengue management** |  |  |  |  |  |  |
| **2** | **Personal leadership ability of your community** |  |  |  |  |  |  |
| **3** | **Health care provider’s capacity of your area** |  |  |  |  |  |  |
| **4** | **Need assessment for dengue prevention activities of your area** |  |  |  |  |  |  |
| **5** | **Sense of the community as dengue is a problem of your area** |  |  |  |  |  |  |
| **6** | **Leader group networking of your area** |  |  |  |  |  |  |
| **7** | **Communication of dengue information of your area** |  |  |  |  |  |  |
| **8** | **Community leadership on Dengue prevention ( strengths) of your area** |  |  |  |  |  |  |
| **9** | **Religious leader capacity of your area** |  |  |  |  |  |  |
| **10** | **Leader group and community networking of your area** |  |  |  |  |  |  |
| **11** | **Resource mobilization for dengue prevention activities of your area** |  |  |  |  |  |  |
| **12** | **Dengue working group of your area** |  |  |  |  |  |  |
| **13** | **Community participation of your area** |  |  |  |  |  |  |
| **14** | **Continuity of activities on dengue prevention of your area** |  |  |  |  |  |  |

**As a member of dengue management leadership group, Please mention the current situation of your community capacity for dengue management according to your perception**

**Age :………………………………**

**Sex :………………………………….**

**Employment : ……………………………**
