# Supplementary material for: Health seeking behaviours, dengue prevention behaviours and community capacity for sustainable dengue prevention in a highly dengue endemic area, Sri Lanka
Source: BMC Public Health. 2023 Mar 16;23:507. doi: 10.1186/s12889-023-15404-5 (PMC10022255; doi:10.1186/s12889-023-15404-5)
Supplement: Supplementary file 4 — Additional file 4. [file 12889_2023_15404_MOESM4_ESM.docx]

**OPERATIONALIZATION OF THE VARIABLES**

**SOCIO-DEMOGRAPHIC**

| GCE (A/L) passed: General Certificate Examination (Advanced Level) is the high school examination [Qualifying examination for state university entrance] |
| --- |
| GCE (O/L) passed: General Certificate Examination (Ordinary Level) is the secondary school examination which is leading to A/L education in state sector [Qualifying examination for A/L examination] |

**Standard of Living Index (SOLI)**

The Modified SOLI by Munasinghe, 2002 to classify the individual’s social status which was based on demographic and Health survey format worldwide was taken to assess the SOLI of the participants [15].

**Classification of SOLI Amenities /Facilities**

| **Amenities /Facilities** | **Score** | | |
| --- | --- | --- | --- |
|  | **2** | **1** | **0** |
| Electricity |  | Yes | No |
| Solar power/ Generator/ battery | Solar power | Generator/ battery | None |
| Radio / TV | Both | One | None |
| Mobile phone/ land phone | Both | One | None |
| Refrigerator |  | Yes | No |
| Ownership of a vehicle | A car/ Van/ Bus/  Other vehicle | Three wheel/ motor bike/ bicycle | None |
| Toilet facilities | Flush water seal | Pit/ other | None/Natural |

The total score was classified in to following three categories and two categories of 6 or less and more than 6.

| **SOLI** | **SCORE** |
| --- | --- |
| High | 9 – 12 |
| Medium | 5 - 8 |
| Low | ≤4 |

**MAIN VARIABLES**

1. **HEALTH SEEKING BEHAVIOUR**

The health seeking behaviours were enquired through close ended questions. There were pre-coded options for each item of the questioner. If any of the mentioned options were not relevant, respondents were asked to specify their action for each question. Total part was given 30 marks according to the marking scheme and converted into percentage (Range 0 – 100%). The overall HSB was categorized in to two groups as “adequate” and “inadequate”. The “adequate behaviour” was described as taking ≥50% of total score. Total part of the health-seeking behaviour for dengue prevention was given 100 marks.

1. **DENGUE PREVENTION BEHAVIOR**

The DPB assessment was consisted of five parts. It was evaluated using ‘waste management (25 marks), outdoor water container management (10 marks), indoor water container management (10 marks), roof gutter management (10 marks) and water storage management (10 marks) which were observed by the interviewers. Waste management was assessed by 10 broad areas covering the 3R concept (Reduce, Reuse and Re-cycling). Total part of the DPB was given 65 marks and the percentage was taken for the overall prevention behaviour. The overall DPB was categorized in to two groups as “adequate” and inadequate”. The “adequate behaviour” was described as taking ≥50% of total score.

1. **COMMUNITY CAPACITY**

Modified Dengue Community Capacity Assessment Tool (DCCAT) was used to assess the perceived capacity of the community on dengue prevention. It has 14 key items, which are “critical situation management, personal leadership, health care provider capacity, needs assessment, senses of community, leader group networking, communication of dengue information, community leadership, religious capacity, leader group and community networking, resource mobilization, dengue working group, community participation, and continuing activities” [23]. It was measured by five-point Likert scale which was categorized in to “Very High (5)”, “High (4)”, “Moderate (3)”, “Low (2)”, and “Very low (1)” capacity groups. Zero marks were given for the “Not sure” answers. The numbers and the percentages for each item were described according to the categories of Likert scale (5-4-3-2-1-0). The scale was given zero to 70 marks for the tool and percentage was taken for each tool. Out of total, ≥50% was considered as an “adequate community capacity”.
